# Sexual behavior and reproductive health among HIV infected adolescents in RBC, IHDPC, Clinic during 2011 therapeutic holidays

**DOI:** 10.1186/1742-4690-9-S1-P102

**Published:** 2012-05-25

**Authors:** Ange Anitha Irakoze, Diane Tuyishimire, B Ami Bugingo, Sabin Nsanzimana, M Josee Maliboli, Melanie Muhizi, Angelique Nkuliza, Simon Niyonsenga, M Gasana, Ciprien Baribwira, Jackson Sebeza, Laetitia Umulisa

**Affiliations:** 1 Rbc, Ihdpc, Hiv Division.Former Trac Plus, Kigali, Rwanda

## Background

Access to SRH services for adolescents in general and for HIV infected specifically is still challenging , resulting in low level of awareness , unsafe sex practices, risk of pregnancies, STI , contamination or super infection.

## Objectives

To determine the patterns of sexual behavior, reproductive health among HIV infected adolescents followed at IHDPC/RBC HIV clinic.

## Methods

During 2011 therapeutic holidays for HIV adolescents followed in RBC/IHDPC/HIV Clinic, 151 of them (73 girls and 78 boys) were assessed about sexual behavior and SRH using a modified HEEADS assessment self-administrated questionnaire and interviews. Parental permission was sought.

## Results

The mean ages (+/-SD) of the girls and boys were 16.3 +/- 0.165 years.

About disclosure: 8.7% of the adolescents were not willing to disclose their HIV status to their sexual partners, yet 76% of interviewed have a partner.

About safe sex: Overall, 8.4% of adolescent are sexually active all above 15years (4.4% of the girls and 12% of the boys). Boys are three times more sexually active than girls and 66.7.1% of sexually experienced boys had used condoms. Among sexually experienced girls only 33.7% used condoms, surprisingly 96.6% of interviewed declared not willing to have protected sex.

About Contraception and pregnancy: among sexually experienced girls 36.7%, had ever used contraception and prevalence of adolescent pregnancy was 2.6%.

Information about sexuality: 81.12% of adolescents have ever learnt about sexuality (51.7 % aged 15-19 years), 49.6% can discuss sexual issues, among them 55% are girls and 45%; are boys.

## Conclusion

In this adolescent cohort, sexual activity seems to debut after 15 years old, safe sex practice is not optimal, and contraception among sexual actives girls is low and information level about SRH need to be improved, underling the urgent need of implementing SRH program for HIV adolescents.

